# Supplementary figures and images for: Multifarious Translational Regulation during Replicative Aging in Yeast
Source: J Fungi (Basel). 2022 Sep 5;8(9):938. doi: 10.3390/jof8090938 (PMC9500732; doi:10.3390/jof8090938)

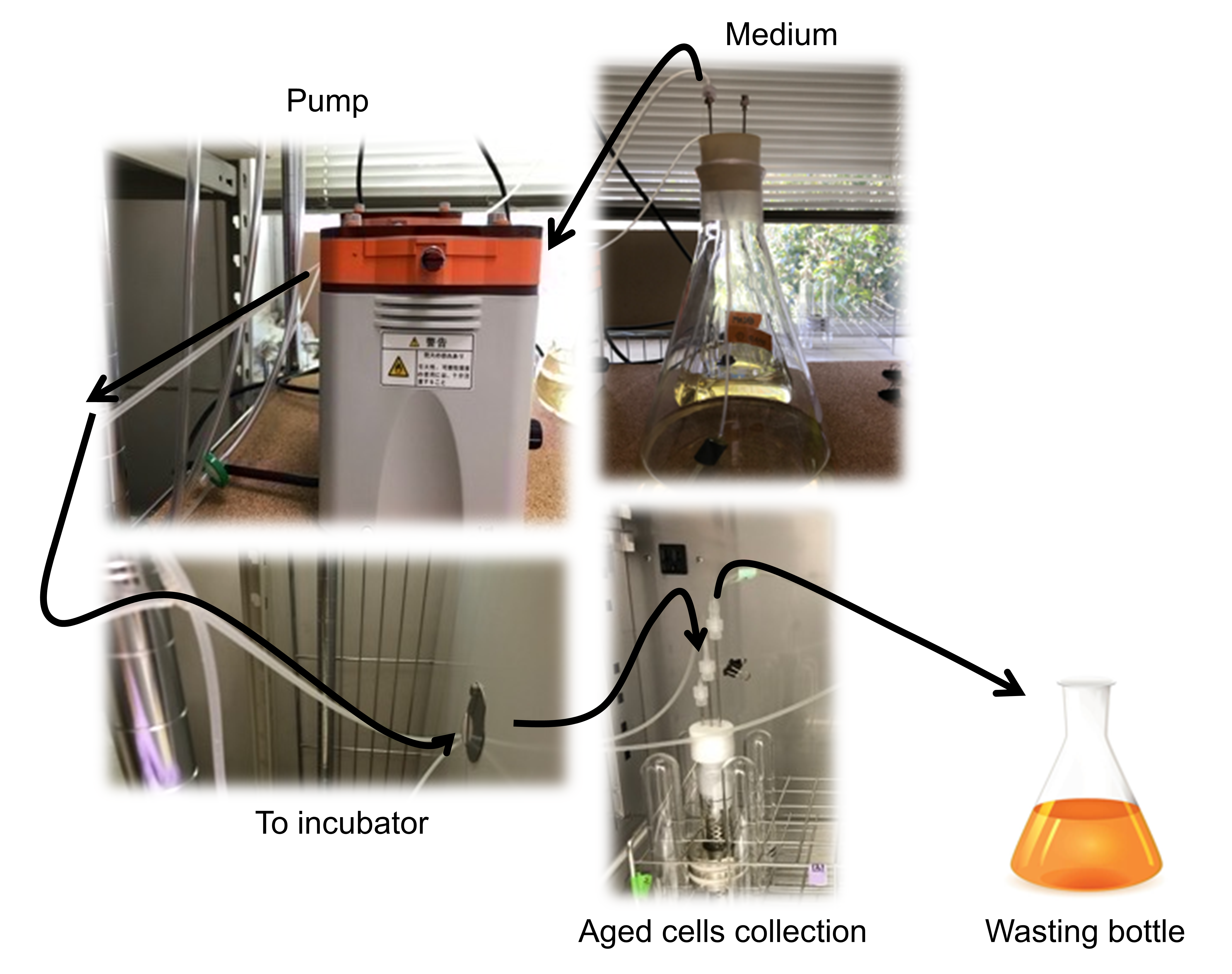

Supplement: Supplementary file 1 [file jof-08-00938-s001.zip › Supplementary Files/Figure S1.png]

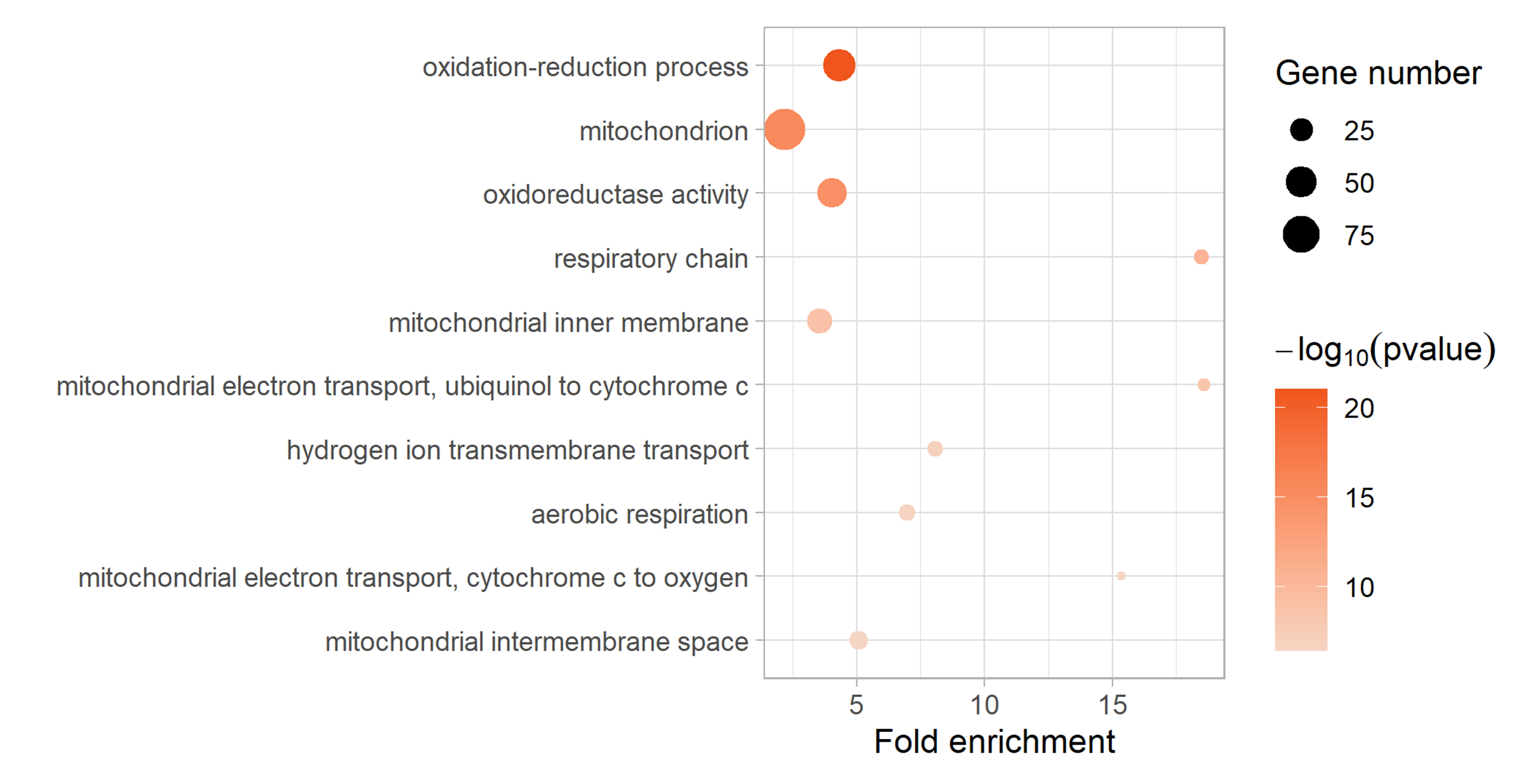

Supplement: Supplementary file 1 [file jof-08-00938-s001.zip › Supplementary Files/Figure S2.png]

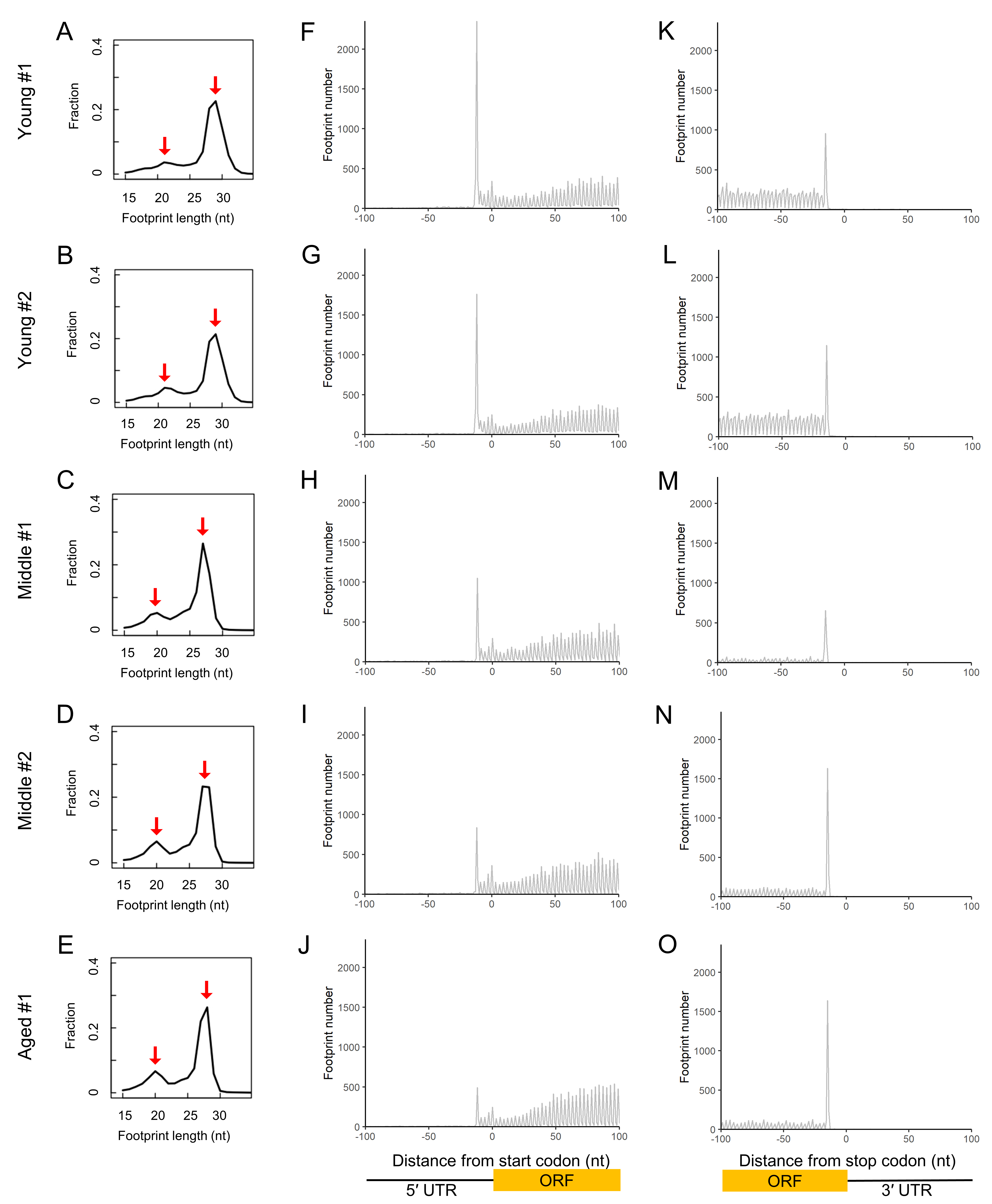

Supplement: Supplementary file 1 [file jof-08-00938-s001.zip › Supplementary Files/Figure S3.png]

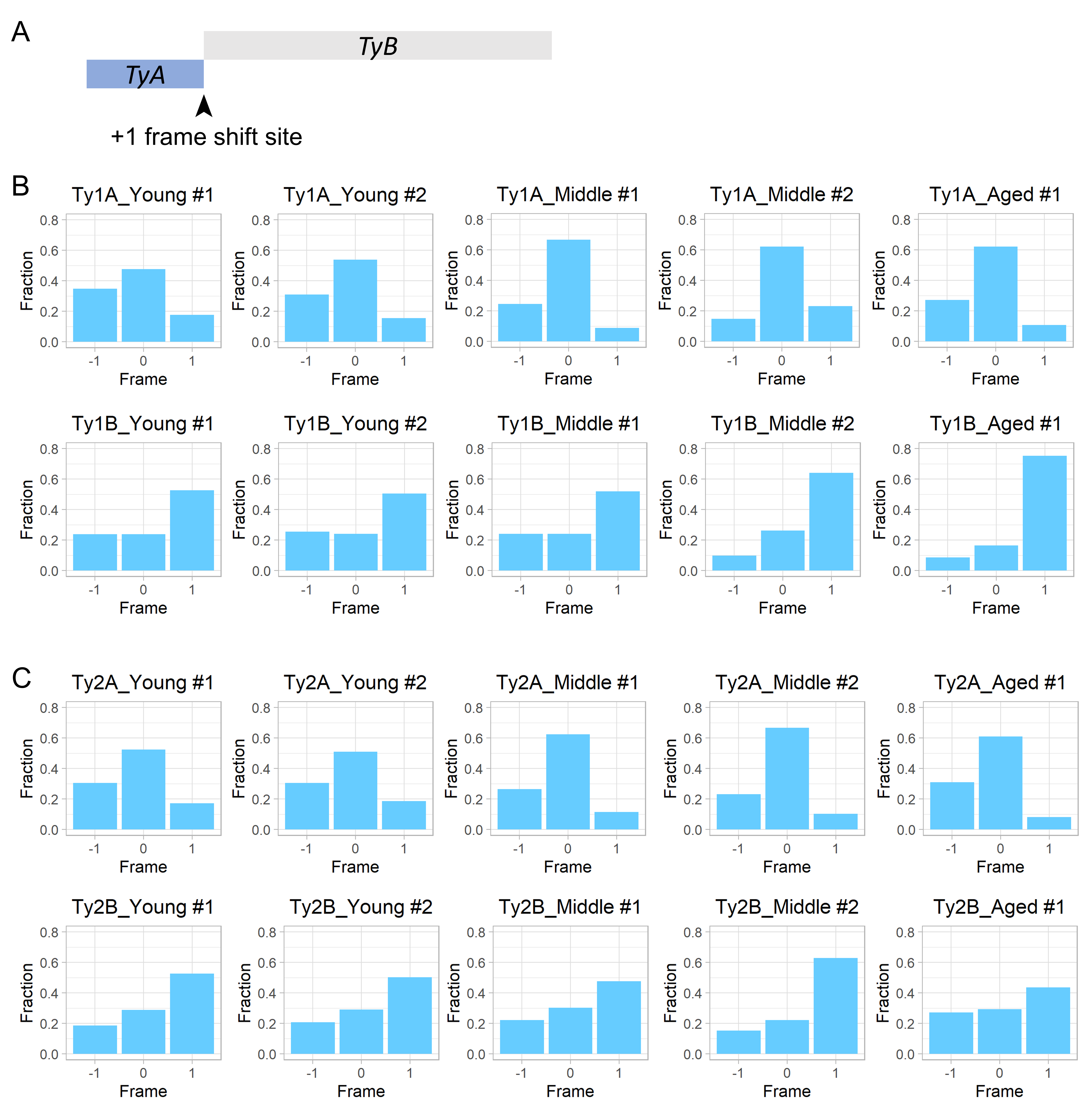

Supplement: Supplementary file 1 [file jof-08-00938-s001.zip › Supplementary Files/Figure S4.png]

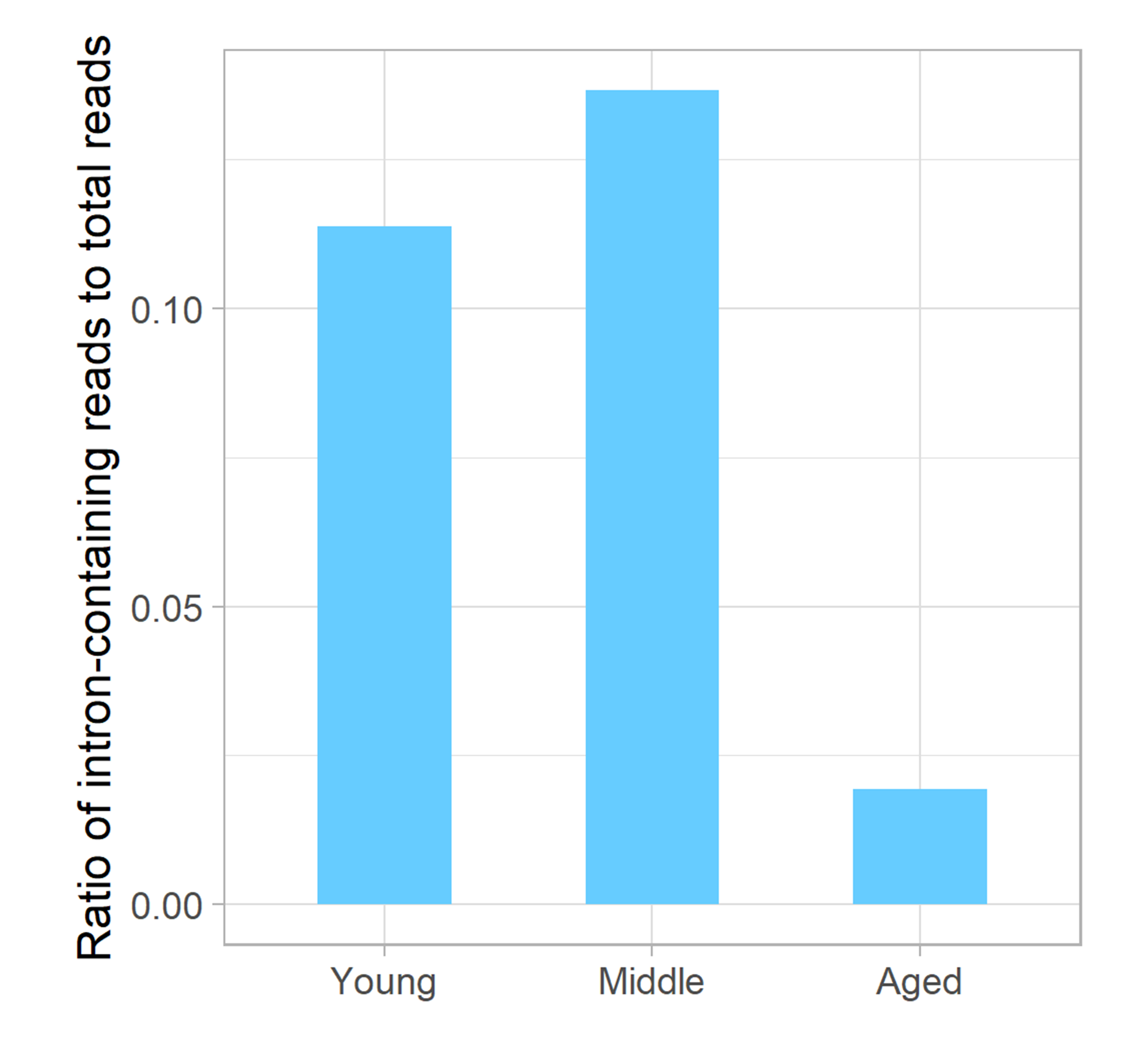

Supplement: Supplementary file 1 [file jof-08-00938-s001.zip › Supplementary Files/Figure S5.png]
